# Immunologic and Genetic Contributors to CD46-Dependent Immune Dysregulation

**DOI:** 10.1007/s10875-023-01547-y

**Published:** 2023-07-21

**Authors:** Benedikt J Meyer, Natalia Kunz, Sayuri Seki, Rebecca Higgins, Adhideb Ghosh, Robin Hupfer, Adrian Baldrich, Julia R Hirsiger, Annaïse J Jauch, Anne-Valérie Burgener, Jonas Lötscher, Markus Aschwanden, Michael Dickenmann, Mihaela Stegert, Christoph T Berger, Thomas Daikeler, Ingmar Heijnen, Alexander A Navarini, Christoph Rudin, Hiroyuki Yamamoto, Claudia Kemper, Christoph Hess, Mike Recher

**Affiliations:** 1 Immunodeficiency Laboratory, Department of Biomedicine, University Hospital Basel, Basel, Switzerland; 2 Immunobiology Laboratory, Department of Biomedicine, University Hospital Basel, Basel, Switzerland; 3 Complement and Inflammation Research Section, CIRS, DIR, NHLBI, NIH, Bethesda, USA; 4 AIDS Research Center, National Institute of Infectious Diseases, Tokyo, Japan; 5 Dermatology, University Hospital Basel, Basel, Switzerland; 6 Competence Center for Personalized Medicine, University of Zürich/Eidgenössische Technische Hochschule (ETH), Zürich, Switzerland; 7 Translational Immunology, Department of Biomedicine, University Hospital Basel, Basel, Switzerland; 8 Department of Angiology, University Hospital Basel, Basel, Switzerland; 9 Clinic for Transplantation Immunology and Nephrology, University Hospital Basel, Basel, Switzerland; 10 Rheumatology Clinic, University Hospital Basel, Basel, Switzerland; 11 University Center for Immunology, University Hospital Basel, Basel, Switzerland; 12 Division Medical Immunology, Laboratory Medicine, University Hospital Basel, Basel, Switzerland; 13 University Children’s Hospital, University of Basel, Basel, Switzerland; 14 Cambridge Institute of Therapeutic Immunology & Infectious Disease, Department of Medicine, University of Cambridge, Cambridge, UK

**Keywords:** Inborn errors of immunity, primary immunodeficiency complement, CD46, haploinsufficiency, atypical hemolytic uremic syndrome, systemic lupus erythematosus, aHUS, SLE, next-generation sequencing, penetrance

## Abstract

Mutations in *CD46* predispose to atypical hemolytic uremic syndrome (aHUS) with low penetrance. Factors driving immune-dysregulatory disease in individual mutation carriers have remained ill-understood. In addition to its role as a negative regulator of the complement system, CD46 modifies T cell-intrinsic metabolic adaptation and cytokine production. Comparative immunologic analysis of diseased vs*.* healthy CD46 mutation carriers has not been performed in detail yet. In this study, we comprehensively analyzed clinical, molecular, immune-phenotypic, cytokine secretion, immune-metabolic, and genetic profiles in healthy vs*.* diseased individuals carrying a rare, heterozygous *CD46* mutation identified within a large single family. Five out of six studied individuals carried a *CD46* gene splice-site mutation causing an in-frame deletion of 21 base pairs. One child suffered from aHUS and his paternal uncle manifested with adult-onset systemic lupus erythematosus (SLE). Three mutation carriers had no clinical evidence of CD46-related disease to date. CD4^+^ T cell-intrinsic CD46 expression was uniformly 50%-reduced but was comparable in diseased vs*.* healthy mutation carriers. Reconstitution experiments defined the 21-base pair-deleted *CD46* variant as intracellularly—but not surface-expressed and haploinsufficient. Both healthy and diseased mutation carriers displayed reduced CD46-dependent T cell mitochondrial adaptation. Diseased mutation carriers had lower peripheral regulatory T cell (Treg) frequencies and carried potentially epistatic, private rare variants in other inborn errors of immunity (IEI)-associated proinflammatory genes, not found in healthy mutation carriers. In conclusion, low Treg and rare non-*CD46* immune-gene variants may contribute to clinically manifest *CD46* haploinsufficiency-associated immune-dysregulation.

**Supplementary Information:**

The online version contains supplementary material available at 10.1007/s10875-023-01547-y.

## Introduction

CD46 (membrane cofactor protein, MCP) is a type I transmembrane protein expressed on nearly all human nucleated cells and capable of binding specifically to the complement activation fragments C3b and C4b [1]. The *CD46* gene is located within a cluster of complement regulatory genes at chromosome 1q3.2 [2]. CD46 and other complement regulatory proteins contain multiple repeats of a complement control protein domain (CCP) that contains binding sites for complement C3b, C4b, and/or factor I [3]. CD46 possesses cofactor activity in that it binds factor I, a plasma serine protease that proteolytically inactivates CD46-bound C3b and C4b. In addition to its co-factor activity, CD46 has important immune-modulatory activity. CD46 co-stimulation not only enforces IFN-γ production of CD4^+^ T helper cells and T helper type 1 (Th1) cell differentiation, but also mediates the Th1 contraction program, driving induced-Treg (iTreg) differentiation [4, 5]. The latter is enforced by the T cell activation-induced recruitment of CD46 to the immune synapse requiring its serine- threonine- and proline-rich STD region [6]. The initial Th1 induction by CD46 is controlled by an interaction with the Notch-1 ligand Jagged-1 [7]. CD46 has also important CD8^+^ T cell intrinsic functions guaranteeing normal IFN-γ secretion and cytotoxicity [8]. CD46, engaged by autocrine C3b, controls immune-metabolic adaptations during T cell activation, such as induction of the amino acid transporter LAT1, the glucose transporter GLUT1 and LAMTOR5 which assembles the amino acid sensing Ragulator-Rag-mTORC1 complex [9]. There is no expression of CD46 in somatic tissues of rodents, including mice [10], precluding the analysis of *in vivo* consequences of human CD46 mutations in the murine system.

aHUS is a life-threatening, acute disease characterized by flares of thrombotic microangiopathy (TMA) in the kidney and other organs related to the uncontrolled activation of the alternative complement pathway [11–18]. Rare germline mutations in several complement proteins including regulatory factors predispose to aHUS via loss- or gain-of function mechanisms (Table 1).

**Table 1:** Inborn errors of complement-related genes causing atypical hemolytic uremic syndrome

Gene	*Inheritance*	Functional consequnce of mutation	Proportion among atypical HUS patients
*CFHR1/CFHR4 *deletion	*AR*	Autoantibodies inactivating CFH	26.5%
*CFH*	*AD*	LOF	20%
*CD46*	*AD*	LOF	10%
*CFI*	*AD*	LOF	6%
*C3*	*AD*	GOF	5%
*CFH/CFHR1 *hybrid allele	*AD*	LOF	3–5%
*THBD*	*AD*	LOF	2–5%
*CFB*	*AD*	GOF	2%

Proportions are derived from a recently updated gene review [14]

Heterozygous loss-of-function human *CD46* mutations have been demonstrated to account for 5–20% of all aHUS cases with high rates of recurrence after the first flare [19]. The complement C5-inhibiting monoclonal antibody (mAb) eculizumab is effective in treatment of aHUS underpinning that complement activation is mediating the disease [20]. However, not all *CD46* mutations linked to aHUS were located in its extracellular domain, implying that the intracellular signaling cascade regulating the metabolism of T (and possibly other) cells might be involved in aHUS disease [3]. The clinical penetrance in carriers of disease-associated *CD46* mutations is 30–50% [3, 21], the reason for this being insufficiently understood. Known triggers inducing aHUS flares in individuals carrying predisposing mutations are inflammation (infections or vaccinations), medication (e.g., cyclosporine), malignancy, pregnancy and transplantation [22]. CD46 mutations have also been—albeit less consistently—linked to other immune-dysregulatory diseases, such as systemic lupus erythematosus (SLE) [3], common variable immunodeficiency (CVID)-like antibody deficiency [7] as well as pregnancy-related preeclampsia [3].

To date, no in-depth immunologic analysis including CD46 gene/protein regulation in mutation carriers within one family has been performed, which we aimed for in the present study.

## Methods

### Study of Human Subjects

The work was carried out in accordance with the Declaration of Helsinki for experiments involving human subjects. All tested individuals were following informed consent enrolled into our prospective cohort study of the functional and genetic architecture of primary immunodeficiencies, which has been approved by the ethical committee of northwestern and central Switzerland (EKNZ 2015-187).

### Diagnostic Immunophenotyping and Serum Complement Activity Assays

Serum immunoglobulin levels and lymphocyte subpopulations were assessed as previously described [23]. Functional assessment of specific complement pathways in serum was performed using a well validated ELISA-based format [24].

### Genomic DNA Isolation and cDNA Synthesis from RNA

Genomic DNA was isolated from cultured PHA T cell blasts using the QIAmp DNA blood Mini Kit. RNA was isolated from peripheral blood mononuclear cells (PBMCs) using Trizol (Thermo Fisher Scientific) for cell lysis. Chloroform (Sigma-Aldrich) was added and lysates were centrifuged for 15 min at 4°C 14’000 g according to the manufacturer’s protocol. RNA was purified with the QIAamp RNA Blood Mini Kit (Qiagen). RNA concentration was determined using the NanoDrop 2000c (Thermo Fisher Scientific). DNA was digested with DNase I (Promega). 100 ng/μl–1 μg/μl of RNA were used for cDNA synthesis. Random primers (Promega) were annealed at 70°C for 5 min. cDNA synthesis was performed according to the Qiagen’s GoScript Reverse Transcription System protocol in a TProfessional TRIO PCR Thermocycler (Core Life Sciences, Laguna Niquel, CA, USA).

### End-Point PCR

Endpoint PCR was performed in a TProfessional TRIO PCR Thermocycler using the GoTaq G2 Polymerase (Promega) with a primer concentration of 0.5 μM and using DNA input template of 50–100 ng / 50 μl PCR reaction. PCR products were separated on a 1.5% agarose gel for the genomic DNA PCR or on a 2.8% agarose gel for the cDNA PCR. PCR bands were cut and DNA was isolated using the Quiaquick Gel Extraction Kit (Qiagen). Sanger sequencing was performed by Microsynth (St.Gallen, Switzerland). In CD46 mutation carriers, there was a 21-nucleotide deletion (GTAAGCCCCCAATATGTGAAA) detected skipping part of exon four.

### Real-Time PCR

Real time PCR was performed using the syber green-based GoTag qPCR Master Mix (promega) on an Applied Biosystem ViiA 7 real time PCR machine with 0.5 μM primer concentration. Gene expression was normalized to *ACTB*/*GAPDH* using the calculation method designed by Michael W. Pfaffl [25].

### PCR Primers

All primers were designed using the NCBI primer blast web tool (https://www.ncbi.nlm.nih.gov/tools/primer-blast/) and ordered as DNA oligos from Microsynth. The forward primer of the wild type (WT) and mutant *CD46* real-time primer pair was set into the deleted part of exon 4.

Genomic DNA PCR detecting *CD46* mutation:

Fw: AAGAAACCACCCCCTCAAACTA
Rev: CTCGGTGCTAGTTAAGAAATCCT

cDNA PCR detecting *CD46* deletion:

Fw: GGAGCCACCAACATTTGAAGC
Rev: CAGACAATTGTGTCGCTGCC

Primers for real-time PCR:

Wild type *CD46:*

Fw: AGCGGTAAGCCCCCAATATG
Rev: TCCAGGTGCAGGATCACAAC

Mutant *CD46*:

Fw: AGTAGCAATTTGGAGCGAGG
Rev: TCCAGGTGCAGGATCACAAC

*ACTB*:

Fw: CGA GCA CAG AGC CTC GCC TT
Rev: CAT CAT CCA TGG TGA GCT GGC G

*GAPDH*:

WT: CCATGAGAAGTATGACAACAGCC
Rev: GGGTGCTAAGCAGTTGGTG

### Antibodies and Reagents

Antibodies to CD3 (OKT-3) and CD46 (TRA-2-10) for CD4^+^ T cell in vitro stimulation were kindly provided by Claudia Kemper (NHLBI/NIH). Agonistic anti-human CD28 (CD28.2) for co-stimulation was purchased from BD (Franklin Lakes, NJ, USA). Anti-human antibodies for flow cytometry are listed in the corresponding section. Mitotracker red/green/deep red dyes were purchased from Thermo Fisher (Waltham, MA, USA). Compounds for mitochondrial perturbation (oligomycin, FCCP and rotenone) were obtained from Sigma Aldrich (St. Louis, MO, USA).

### CD4^+^ T Cell Purification and In Vitro Activation

PBMCs were isolated from freshly-drawn blood by centrifugation on after layering onto Lymphoprep separation medium (Corning, Vienna, VA). CD4^+^ T cells were then enriched by MACS separation using the MACS human CD4^+^ Positive T cell Isolation Kit (Miltenyi Biotech, Bergisch Gladbach, Germany) according to the manufacturer’s instructions. Purity of isolated CD4^+^ T lymphocytes was typically > 97%. Purified CD4^+^ T cells were either analyzed directly after isolation (ex vivo) or activated for 36 h in 48-well culture plates (Greiner, Monroe, NC) at a density of 2.5–5.0 × 10^5^ cells/well in media containing 50 U/ml recombinant human IL-2 at 37^o^C and 5% CO2. 2 μg/ml stimulating antibodies (anti-CD3, anti-CD28 or anti-CD46 in the depicted combinations) were immobilized overnight at 4^o^C on culture plates before CD4^+^ T cell plating.

### Flow Cytometry

Freshly purified or stimulated CD4^+^ T cells were washed twice with FACS buffer, stained with a PE-conjugated anti-CD46 antibody (8E2, 1:50) (eBioscience/Thermo Fisher, San Diego, CA, USA) for 15 min at room temperature and washed twice with FACS buffer. For binding specificity mapping, PBMCs were stained similarly with anti-CD46-PE [TRA-2-10 (Biolegend, San Diego, CA, USA), E4.3 (BD), 8E2 (eBioscience), 1:100], anti-CD46-FITC (MEM-258, Biolegend, 1:100) or via a two-step stain of anti-CD46 GB24 mAb (10 μg/ml, kindly provided by Claudia Kemper) and rat anti-mouse IgG1-FITC (RMG1-1, 1:100, Biolegend). Mouse IgG1 isotype-PE (3.6.2.8.1, eBioscience; MOPC-21, Biolegend) and mouse IgG2a-PE (clone G155-178, BD) were used for isotype control staining. For *ex vivo* activation marker analysis, cells were laid in culture for 36 h and surface-stained with anti-CD3-Brilliant Violet 510 (UCHT1), anti-CD4-APC (SK3), anti-CD8-Brilliant Violet 421 (SK1), anti-4-1BB-PE-Cy7 (4B4-1) (from Biolegend), Fixable Viability Dye eFluor 780 and anti-OX40-PE (ACT-235) (from eBioscience).

Intracellular FoxP3 staining was performed on PBMCs with eBioscience FoxP3/transcription factor staining kit (eBioscience/Thermo Fisher) with anti-human FoxP3-PE (PCH-101) and rat IgG2a isotype control-PE (eBR2a) (from eBioscience), with prior surface co-staining of anti-CD127-APC (A019D5), anti-CD25-FITC (BC96), anti-CD8-Brilliant Violet 421 (SK1), anti-CD4-PE-Cy7 (SK3) and anti-CD3-Brilliant Violet 510 (UCHT1) (from Biolegend).

For mitotracker staining, freshly purified or stimulated CD4^+^ T cells were resuspended in 100 μl pre-warmed FACS buffer and 100 μl warm staining solution was added (FACS buffer containing mitotracker red or green and mitotracker deep red stain (100nM each) [23]. Cells were stained for 20 min at 37^o^C, then washed 2 × with complete medium and 1 × with FACS buffer.

Samples were subsequently resuspended in FACS buffer and analyzed on the Accuri C6 cytometer (BD) or Cytoflex LX (Beckman Coulter, Brea, CA, USA). Data analysis was conducted using the FlowJo 10.0.8 software (FlowJo).

### CD46 Protein Structure Prediction

A brouser-adapted Alphafold2 (ColabFold v1.5.2) [26] -based structure prediction of del21bp *CD46*-translated product was performed on 30 March 2023 with NP_758861.1 membrane cofactor protein isoform 3 precursor [*Homo sapiens*] utilized as the WT CD46 query.

### Cytokine Measurements

Accumulated cytokine levels in the supernatant of purified CD4^+^ T cells stimulated for 36 h were assessed using the LEGENDplex human Th1 panel (Biolegend) according to the manufacturer’s instructions and analyzed on the Accuri C6 cytometer (BD). Data analysis was conducted using the FlowJo 10.0.8 software (FlowJo).

### CD46 Reconstitution Assay

A transient CD46 overexpression of 21 base pair-deleted (del21bp) *vs.* WT *CD46* GFP reporter/CMV-promotor pRP[Exp]-EGFP/Puro-CAG>hCD46[NM_172359.2] vectors (VectorBuilder, Chicago, IL, USA) was performed on a *CD46*-knocked out human myeloid HAP-1 cell line (Horizon Discovery, Cambridge, UK). Cells were maintained in Iscove's Modified Dulbecco's Medium (IMDM) (Gibco/Thermo Fisher) supplemented with 10% fetal bovine serum (FBS) (Cytiva, Marlborough, MA, USA) and 1% penicillin/streptomycin (Gibco/Thermo Fisher). To obtain the del21bp *CD46* vector, the WT plasmid was subjected to KOD-plus-mediated site-directed mutagenesis (Toyobo, Osaka, Japan) and isolated for single colonies with the following primers:

Fw: 5'-CAGTAGCAATTTGGAGCGAGGTTTTGTGTACACCAC-3'
Rev: 5'-GTGGTGTACACAAAACCTCGCTCCAAATTGCTACTG-3'

The designed mutant plasmid was purified with Endofree Maxi plasmid purification kit (Qiagen, Hilden, Germany) and Sanger-sequenced for the full *CD46* region to confirm correct mutagenesis. 1.0 × 10^5^ of *CD46*-knocked out HAP-1 cells were transfected with WT and/or del21bp *CD46* vectors using Lipofectamine 3000 (Thermo Fisher) in 24-well flat-bottomed plates (Falcon/Corning) for 24 hours, and subsequently stained with anti-human CD46-PE (clone E4.3) for surface and intracellular expression analysis. Intracellular staining was performed with Cytofix/Cytoperm kit (BD). Samples were analyzed on FACS Lyric cytometer (BD) and data were analyzed with FlowJo 10.0.8 software (BD/FlowJo).

### Measurement of Oxygen Consumption Rate (OCR) and Extracellular Acidification Rate (ECAR)

Stimulated CD4^+^ T cells were resuspended in serum-free unbuffered RPMI-1640 medium (Sigma Aldrich) and were plated onto Cell-Tak-coated (Corning, Reinach, Switzerland) XF96 Seahorse plates (Seahorse Bioscience, North Billerica, MA, USA) in triplicates at a density of 2.5x10^5^ viable cells per well. The Seahorse cartridge injection ports were loaded with oligomycin (1 μM), Carbonyl cyanide-4-(trifluoromethoxy) phenylhydrazone (FCCP, 2 μM) or rotenone (1 μM—all from Sigma Aldrich), respectively, and metabolic profiles were measured on a Seahorse XF96 Extracellular Flux analyzer (Seahorse Bioscience).

Metabolic parameters were calculated as follows:

1. basal OCR (total OCR, mitochondrial and non-mitochondrial) = OCR before mitochondrial perturbation
2. basal respiration = basal/total OCR – OCR after rotenone injection
3. Non-mitochondrial respiration = OCR after rotenone injection
4. ATP coupled respiration = basal/total OCR – OCR after oligomycine injection
5. Leak respiration = OCR after oligomycine injection – OCR after rotenone injection
6. maximal respiratory capacity = OCR peak after FCCP injection – OCR after rotenone injection
7. basal ECAR = initial ECAR before mitochondrial perturbation
8. maximal ECAR = ECAR peak after mitochondrial perturbation

### Whole Exome Sequencing

Whole exome sequencing has been performed as recently described [23]. Rare immunodeficiency associated mutations were screened using the list of primary immunodeficiencies that has been recently reported [27]. All reported variants have been confirmed by genome viewer. Immunodeficiency-related gene variants were listed when the CADD Score was >15 and the minor allele frequency listed in gnomad (https://gnomad.broadinstitute.org) was <0.01. All listed variants had a sequencing coverage >25 with the exception of the *TNFRSF4 and STXBP2* variants, where the coverage was <20 but the heterozygous variants were confirmed by integrated genome viewer (IGV).

### NIHR BioResource Rare Diseases Project Database Analysis

The NIHR Bioresource Rare Disease project has provided whole genome sequencing data for more than 10’000 individuals with rare diseases [28]. We selected the rare exonic variants in the whole genome sequencing (WGS) data set of 13’037 individuals in a pre-defined set of PID/IEI genes [29], which were present in participants carrying rare CD46 variants, defining rare as absent from gnomAD at frequencies above 1/1,000 in all major populations, and selecting only variants having a CADD Phred score of at least 15. The listed CD46 variants have been linked to CD46 dependent human disease [15].

### Statistical Analyses

Two-tailed unpaired *t* tests and one-way analysis of variance (ANOVA) were performed on Prism 8.4.3 (San Diego, CA, USA) with a significance value of *p* < 0.05.

## Results

### Heterozygous *CD46* c.475+1G>A Splice-Site Variant in a Large Family with aHUS and SLE-Diseased Individuals

**Fig. 1:**
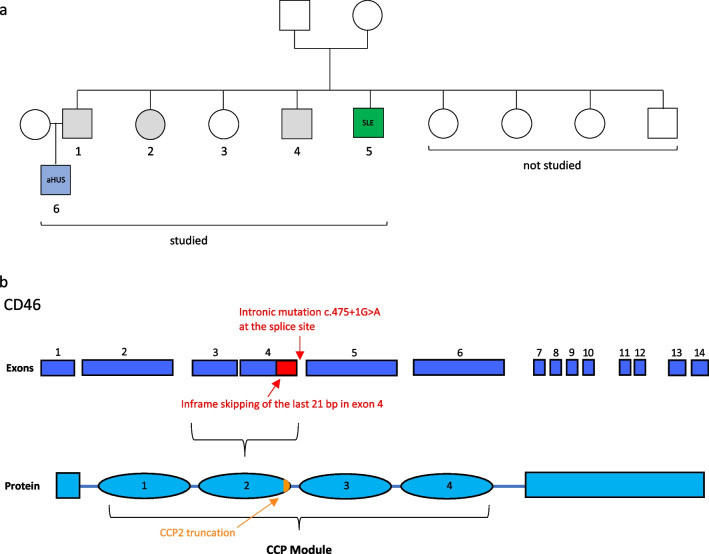
Study design and analyzed *CD46* c.475+1G>A mutation. **a** Visualization of the family tree with several family members carrying a rare *CD46* mutation. Circles indicate females, squares indicate male individuals. The numbered individuals [1–6] have been analyzed in the current study. The blue colored patient manifested with pediatric-onset aHUS [6], and the green colored individual has the diagnosis of adult-onset SLE [5]. Individuals with grey color carry the mutation but do not manifest CD46 associated immune-dysregulation up to date. **b** Exons of genomic *CD46* schematically depicted (deep blue) with corresponding protein modules of CD46 (bright blue). The c. 475+1G>A point mutation is located at the start of the intron between exon 4 and 5, altering a splice site leading to an in-frame truncated exon 4 (skipped exon 4 part in red) lacking in 21 base pairs (bp). The predicted protein variant has a truncated CCP module 2 (residue deletion represented in orange)

**Fig. 2:**
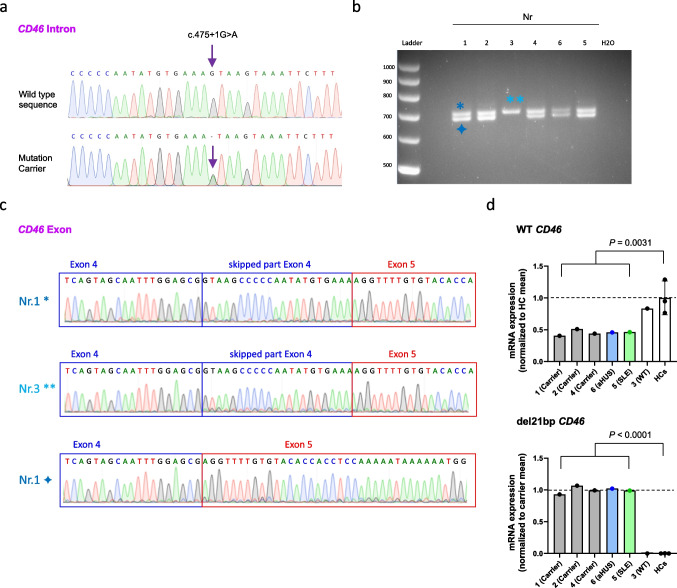
Exon 4 partial skipping and transcript coexistence of mutated *CD46*. **a** Representative Sanger sequencing of a PCR-amplified *CD46*-specific sequence using PBMC-derived genomic DNA. Purple arrow shows the heterozygous *CD46* intronic mutation detected in five out of six tested family members. **b** PCR amplification of a *CD46*-specific sequence using PBMC-derived cDNA revealing two different bands in all tested individuals except family member number 3 which does not carry the *CD46* mutation in genomic DNA. Image shown is zoomed for bands and ladder. **c** Sanger sequencing chromatogram of the bands indicated in **b** show a skipping of 21 nucleotides in the truncated cDNA band from the del21bp *CD46* gene (bottom). **d** mRNA expression quantitation of WT (top) and del21bp mutant (bottom) *CD46* mRNA assessed using PBMC-derived cDNA. Each are normalized as listed on the Y axis. *P* values represent comparison of all five *CD46* mutation carriers and three healthy controls (HCs) by unpaired *t* tests

Whole exome sequencing (WES) analysis identified five individuals from a large family that carried a rare heterozygous *CD46* variant c.475+1G>A (rs753486842) (Figs. 1 and 2). Two of the mutation carriers had a clinical history of a CD46-associated immune dysregulation, while the other three mutation carriers did not develop CD46-associated disease as of today (clinical history, Supplemental Table 1). The pediatric patient, family member Nr.6 (Fig. 1a), had been diagnosed with aHUS at our institution in 2006 at the age of 4 years. The clinical and laboratory characteristics upon admission and of four additional flares of aHUS, all with complete remission, are displayed in Supplemental Figure 1 and Supplemental Table 2. The time interval between aHUS flares was generally increasing, while duration of hospitalization and need of peritoneal dialysis was shortening with each flare (Supplemental Figure 1). A paternal uncle, family member Nr.5 (Fig. 1a), was treated in the adult immunology clinic of our University Hospital due to adult-onset SLE. The main clinical SLE manifestation was an angiopathic ulceration of the left toe associated with elevated anti-phospholipid autoantibodies (Supplemental Figure 2). Other SLE-associated abnormalities were low platelet counts, complement consumption, elevated anti-nuclear autoantibodies and elevated anti-double strand DNA (ds-DNA) autoantibodies (Supplemental Figure 2, Supplemental Table 3). Serum IgM was slightly and polyclonally elevated (Supplemental Figure 2). The SLE patient is one of nine siblings from the same parents. In total, six family members were available for further testing (individuals labeled 1–6, Fig. 1a). The parents and four of their children were not available for the study.

The *CD46* mutation was confirmed by Sanger sequencing in all five family members who carried the mutation based on the WES analysis (Fig. 2a–c). The allele frequency of this mutation at gnomAD (https://gnomad.broadinstitute.org) is 0.000004046 with a CADD score of 23.7—predicting functional relevance. This mutation has been previously reported in a pediatric aHUS patient from Italy [30], without further immunologic or molecular work-up. The mutation leads to alternative splicing on cDNA level with a partial 21 nucleotide deletion of exon 4 (Figs. 1b and 2b+c), which encodes for the CCP2 domain. This mutation-associated partial exon 4 deletion (del21bp) was present at cDNA level in all five mutation carriers (Figs. 1 and 2b+c). Real-time PCR analysis of PBMC-derived cDNA revealed that all five mutation carriers expressed approximately 50%-less *CD46* wild-type mRNA compared to examined healthy controls and the family member that did not carry the mutation (Fig. 2d, top). Mutation carriers exclusively expressed the del21bp *CD46* mRNA (Fig. 2d, bottom).

### CD46 del21bp Mutation Is Loss-of Surface Expression and Lacks Dominant Negativity Regarding Surface Expression of WT CD46

**Fig. 3:**
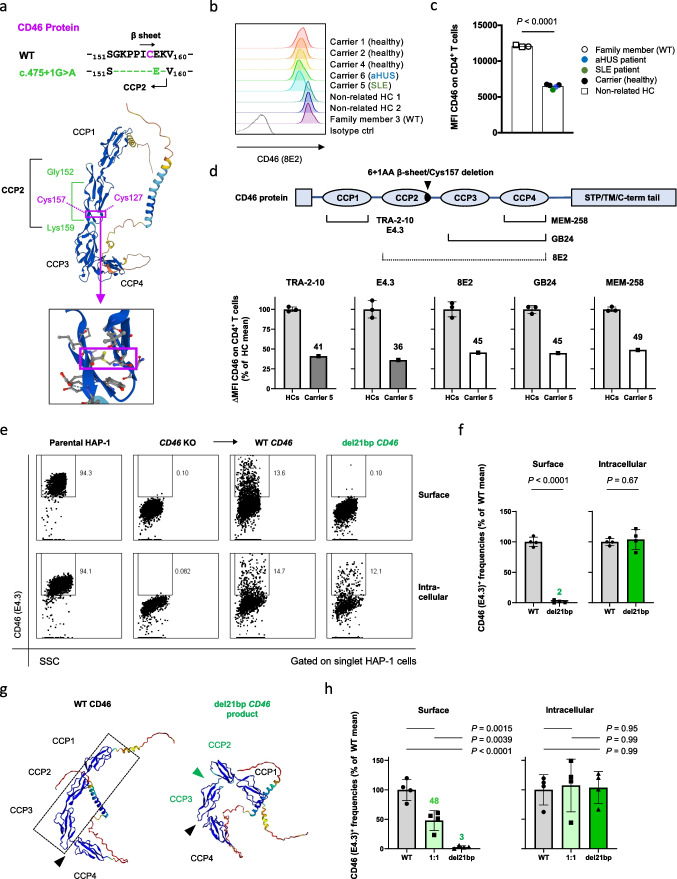
del21bp *CD46* is a loss-of-expression mutation and lacks dominant negativity. **a** Alignment (top) of exon skipping-mediated CD46 protein 6+1-residue deletion flanking glutamic acid (Glu) 158 and its orientation on a reported WT CD46 structure (middle) appended with a magnified view (bottom). Purple boxes show orientation of a CCP domain-conserved disulfide bond between deleted cysteine residue 157 and cysteine residue 127. Structure adopted/downloaded from Alphafold2 (https://alphafold.ebi.ac.uk/entry/P15529). b+c) Flow-cytometric analysis of CD46 surface expression on freshly purified CD4^+^ T cells ex vivo. Representative histogram plots are shown in **b** and MFIs are depicted in **c**. **d** Schematic of CD46-specific monoclonal antibody (mAb) binding sites and CD46 surface binding signal intensity in mutation carrier 5 (SLE) in comparison with unrelated healthy donor controls. Dotted line for 8E2 mAb indicates incomplete epitope identification in literature. Fluorescence deviation values compared with intra-patient isotype control staining are normalized against mean of healthy controls for each. CCP1-specific binding is shown in gray and CCP2/3/4 binding are shown in white for mutation carrier 5. **e** Representative flow-cytometric plot of transient CD46 overexpression via WT versus del21bp *CD46* vectors by transfection of a *CD46*-knocked-out human myeloid HAP1 cell line. **f** Comparison of WT versus del21bp *CD46* protein surface and intracellular expression positivity. Analyzed by unpaired *t* tests. **g** ColabFold v1.5.2-based structure prediction of del21bp *CD46*-variant protein (right) in comparison with queried/predetermined WT CD46 structure (left). Black wedges show a CCP3/CCP4-junction bending contrasting CCP1/CCP2/CCP3 alignment (highlighted in dotted line). Green wedge shows a de novo impairment of CCP2/CCP3. **h** CD46 expression upon WT and del21bp mutant *CD46* co-transfection (1:1 ratio). *P* values represent Tukey’s post hoc multiple comparison tests of one-way ANOVA. Representative of two experiments performed in quadruplicate (f+h)

We next addressed variant CD46 protein encoded by the del21bp *CD46* mutation. The del21bp *CD46* mutation results in a deletion of 6+1 amino acid residues at positions 152-157 and 159 (Gly-Lys-Pro-Pro-Ile-Cys- and Lys), flanking glutamic acid (Glu) residue 158 (Fig. 3a, top). This results in a loss of two conserved prolines at positions 154-155 (PP motif) [31] and, importantly, a β sheet including one of the four universally CCP-conserved cysteines at position 157 building a disulfide bond with cysteine 127 [32–34] (Fig. 3a, bottom). Flow-cytometric analysis showed that both diseased and healthy mutation carriers expressed lower CD4^+^ T cell-intrinsic cell surface CD46 levels compared to healthy controls ex vivo (Fig. 3b+c). A comprehensive CD46-specific antibody surface binding mapping on peripheral CD4^+^ T cells with a set of epitope-mapped [35], commercially available and non-available (GB24) [36] anti-CD46 mAbs in the mutation-carrying SLE patient revealed approximately 50% reduced surface expression (Fig. 3d). This was independent of N-terminal *vs.* C-terminal mAb binding specificity, arguing against intact surface expression of the mutant CD46 protein.

We then performed transient protein overexpression of the del21bp *CD46* variant compared to WT *CD46* in a *CD46*-knocked out human myeloid HAP1 cell line. WT CD46 reconstitution resulted in CD46 surface expression which was absent in the parental cell line (Fig. 3e, columns 1–3 top). In contrast, reconstitution of del21bp *CD46* resulted in a near-complete loss of surface CD46 expression (Fig. 3e, column 4 top), defining the original intronic mutation as a loss-of-expression (Fig. 3f, left). However, intracellular levels of the del21bp CD46 variant were comparable with WT CD46 (Fig. 3e+f). Thus, the del21bp *CD46* variant is expressed as a protein but does not reach the cell surface. This protein trajectory was analogous to a CCP cysteine-to-arginine/glycine substitution described in a case of human complement factor H deficiency [37, 38]. A browser-adapted AlphaFold2-based structure prediction of del21bp *CD46*-translated product suggested an inter-CCP bending, existing only between CCP3/CCP4 in WT [34] (Fig. 3g, left, black wedge), to newly arise between CCP2/CCP3 (Fig. 3g, right, green wedge), losing their near-linear alignment with CCP1 (Fig. 3g, left, dotted box). Co-transfection of WT and del21bp *CD46* at a 1:1 ratio resulted in 50% surface expression compared with WT-only, whereas intracellular expression level was comparable. Thus, the del21bp variant-encoded CD46 protein does not confer dominant negativity regarding surface expression of WT CD46 (Fig. 3h).

### Immune Phenotyping of Diseased vs. Healthy *CD46* Mutation Carriers

**Fig. 4:**
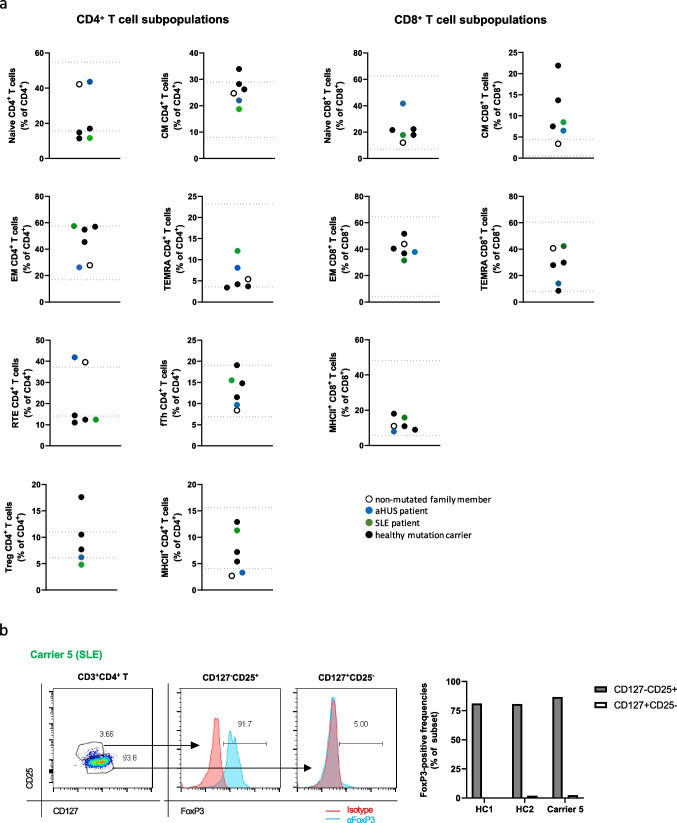
Immune cell subpopulations in heterozygous *CD46* c.475+1G>A mutation carriers. **a** CD4^+^ and CD8^+^ T cell subpopulations as indicated of all tested family members are depicted. The blue and green dots represent the aHUS and SLE patient, respectively. Closed symbols represent mutation carriers while the open symbol represents the family member lacking the mutated allele. The lines mark normal reference values. **b** Left: FoxP3 transcription factor positivity in mutation carrier 5 (SLE) CD4^+^ T cell subpopulations. Right: subpopulation FoxP3 positivity aligned with two unrelated healthy controls. Values are subtracted for positivity in isotype control staining

Next, we performed a detailed phenotypic analysis of lymphocytes, T and B cell subpopulations in all family members using a clinically validated protocol allowing us to link the results to normal in-house reference ranges [23]. Lymphocyte subpopulations were within normal reference ranges for all tested individuals with the exception of the patient with aHUS who showed low-normal CD4^+^ T cells in peripheral blood. Absolute B cells were normal (Supplemental Figure 3). B cell subpopulations were unremarkable with the exception of the patient with SLE who demonstrated high memory and low naïve B cells (Supplemental Figure 3). Four out of five mutation carriers had low naïve CD4^+^ T cells and low recent thymic emigrant (CD45RA^+^CD31^hi^) CD4^+^ T cells (Fig. 4a). The only mutation carrier with normal naïve CD4^+^ T cell frequencies was the patient with aHUS, which plausibly could demonstrate higher naïve CD4^+^ T cells due to the pediatric age at analysis [39] (Fig. 4a). Within the CD8^+^ T cells, five out of five mutation carriers had elevated central memory (CD45RO^+^CD27^+^) subpopulations (Fig. 4a). Intriguingly, while healthy mutation carriers had regulatory CD4^+^CD25^hi^CD127^low^ CD4^+^ T cells (Treg) frequencies within normal reference ranges, both diseased mutation carriers had low Treg (Fig. 4a). CD127^low^CD25^hi^ (surface-defined Tregs) *vs.* CD127^hi^CD25^low^ CD4^+^ T cell populations in mutation carrier 5 (SLE) segregated with intracellular FoxP3 positivity, non-discrepant with two unrelated healthy donors (Fig. 4b), in keeping with literature on Treg surface phenotyping [40, 41]. As tested in the SLE patient, all T-cell subpopulation frequencies were recapitulated when reassessed several years after initial testing, arguing for stable traits (Supplemental Figure 3c). With the exception of slightly elevated IgM in the SLE patient, none of the mutation carriers had dysregulated serum IgG, IgM, or IgA levels (Supplemental Figure 3d). The two main complement pathways were functionally assessed in sera of all tested family members (Supplemental Figure 3e) [24]. The classical complement pathway was functionally unaffected in all family members. Sera from four out of five mutation carriers had activities of the alternative pathway above the reference range, a finding requiring further scrutiny.

### Cytokine Secretion and Immune-Metabolic Profiling of *CD4*^+^ T Cells of Diseased vs. Healthy *CD46* Mutation Carriers

Normal CD46 abundance and activity is required for Th1 and CTL effector activity induction [8, 42]. We thus next measured cytokine production by purified CD4^+^ T cells in all family members plus 4-8 non-related healthy blood donors following T cell receptor ligation alone or in combination with agonistic anti-CD28 or anti-CD46 antibody co-stimulation. T cells of mutation carriers showed a trend to produce equal or more pro-inflammatory cytokines (IL-6, IFN-γ, TNF-α) following T cell receptor ligation plus CD28 co-stimulation compared to controls (Supplemental Figure 4), although these data did not reach statistical significance. This difference was normalized or reversed following CD46 co-stimulation, in keeping with impaired functional CD46 co-stimulation in mutation carriers. IL-10 secretion in CD46 co-stimulated mutation carrier-derived cells was approximately 50%-decreased (*p* = 0.0657) (Supplemental Figure 4), implicating certain involvement in the described immune-dysregulation linked with CD46 haploinsufficiency [5].

**Fig. 5:**
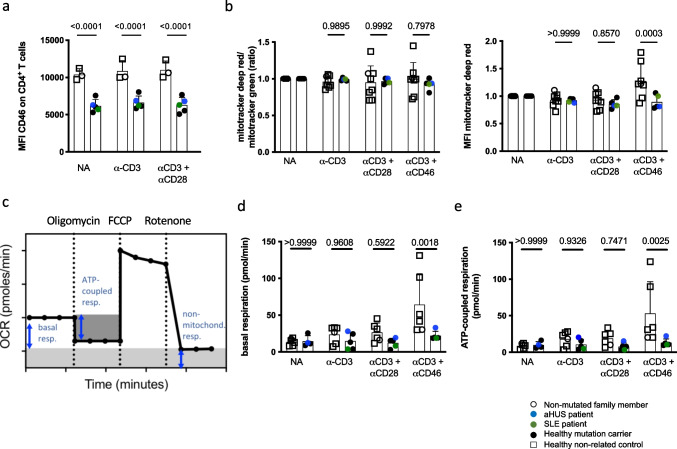
Impaired CD46-dependent immunometabolic adaptation in heterozygous *CD46* c.475+1G>A mutation carriers. **a** CD46 expression (MFI) on CD4^+^ T cells following in vitro stimulation. Purified CD4^+^ T cells were left non-activated (NA) or stimulated with immobilized agonistic antibodies against CD3 or CD3 + CD28 for 36 hours before flow-cytometric analysis. **b** Purified CD4^+^ T cells were either analyzed ex vivo or left non-activated (NA) or stimulated with immobilized agonistic antibodies against CD3, CD3 + CD28 or CD3 + CD46 for 36 h. Flow cytometric analysis of the MFI of mitotracker deep red T cell fluroescence as a marker of mitochondrial membrane potential and the MFI of mitotracker green staining as a marker for mitochondrial mass of CD4^+^ T cells was performed. The ratio of the two MFI’s represents mitochondrial function per mitochondrial mass. **c** An explanatory graph showing cellular oxygen consumption rate and the respiratory indices after perturbation with oligomycin, FCCP and rotenone in the Seahorse flux analyzer. d + e) Quantification of T cell intrinsic oxygen consumption. Purified CD4^+^ T cells were left non-activated (NA) or stimulated with immobilized agonistic antibodies against CD3, CD3 + CD28 or CD3 + CD46. 36 h post stimulation, basal **c** and ATP-coupled **d** oxidative phosphorylation (OXPHOS, OCR) in CD4^+^ T cells was measured on the Seahorse metabolic extracellular flux analyzer. All bars indicate means ± SD. Compared by unpaired *t*-tests as indicated

A major activity of CD46 during T cell activation is the induction of increased nutrient influx and T cell metabolic adaptation [9], including the support of glycolysis and oxidative phosphorylation (OXPHOS). To assess this, we first approximated mitochondrial mass and membrane potential in resting and stimulated conditions in T cells of all family members. As observed ex vivo, CD46 surface expression was lower in mutation carriers following in vitro CD3-only or CD3+CD28 CD4^+^ T cell stimulation (Fig. 5a). No differences were found in mitochondrial parameters directly ex vivo in mutation carriers *vs.* controls (data not shown). However, 36 h following CD4^+^ T cell activation in vitro, the mean fluorescence intensity (MFI) of mitotracker deep red (indicating mitochondrial potential) was significantly lower upon CD46 co-stimulation in mutation carriers, likely reflecting decreased surface CD46 expression (Fig. 5b). Next, the extracellular acidification rate (ECAR), as a surrogate of cellular lactate production (glycolysis), and the oxygen consumption rate (OCR) (OXPHOS) were measured in real-time by metabolic flux analysis in T cells from all family members and controls in the absence or following in-machine T cell activation for 36 h. There was no difference in mutation carriers *vs.* controls in basal or maximal ECAR induced by mitochondrial perturbation (data not shown). In contrast, basal and ATP-coupled respiration (Fig. 5c) were reduced specifically following CD46 co-stimulation in activated T cells of *CD46*-mutated family members (Fig. 5d+e). Maximal respiratory capacity and spare respiratory capacity were not affected by *CD46* mutation status, not even following CD46 co-stimulation (data not shown). In conclusion, the identified CD46 loss-of-expression mutation was directly linked with altered T cell-intrinsic metabolic adaptation following CD46 co-stimulation.

### Private Non-CD46 Rare Germline Variants as Possible Modifiers of Clinical Phenotype and Penetrance in CD46 Mutation Carriers

Penetrance of CD46-dependent aHUS is modified by inflammatory events such as infections or even vaccinations [22, 43, 44], which themselves are shaped in extent by rare variants in genes encoding immune system proteins [45]. To search for possible genetic contributors to penetrance and/or clinical manifestations beyond the CD46 variant, we filtered WES data for rare variants in immune system genes that had been previously linked to IEI [29] (Supplemental Table 4). The aHUS patient carries a heterozygous E148Q variant in the *MEFV* gene, which has been associated with the autoinflammatory Familial Mediterranean Fever [46] and was recently molecularly demonstrated to be gain-of-function regarding inflammasome activation [47]. In addition, the aHUS patient carries a frameshift mutation (CADD Score: 35) in the *NOD2* gene, known to be positively associated with Crohn’s disease [48], another autoinflammatory syndrome (Table 2). This variant is loss-of-function for NOD2 signaling but pro-inflammatory partly due to insufficient NOD2 dependent up-regulation of the NF-kappa B inhibitor A20 (*TNFAIP3*) [49] and inhibits IL-10 transcription further promoting inflammation [50]. None of these two proinflammatory variants were detected in the other four *CD46* mutation carriers (Table 2). The patient with SLE carried a rare heterozygous missense variant in the TNF receptor superfamily co-stimulator *TNFRSF4/*OX40, substituting a signaling peptide residue in the hydrophobic region relatively conserved in mammals (p.R10C) with a CADD score of 22 predicting functional consequences (Supplemental Figure 5 a+b). This variant was not present in the aHUS patient, while being also present in one currently healthy *CD46* mutation carrier (Table 2). CD46 co-stimulation facilitated OX40 expression on CD4^+^ T cells (Supplemental Figure 5c), and ex vivo OX40 expression was increased in CD4^+^ T cells of the SLE patient compared to healthy controls (Supplemental Figure 5d).

**Table 2:** Non-*CD46* rare immunodeficiency-related gene variants filtered in WES

Chr	rsID	Zygo-sity	Gene	cDNA	Amino Acid	Allele Frequency	CADD
Family Member 1 (male, carrier)							
1	rs35012521	het	*NCF2*	c.A1121T	p.N374I	0.005429	28.4
15	rs1352520577	het	*BLM*	c.A3334C	p.N1112H	0.0000088	26.5
Family Member 2 (female, carrier)							
1	rs35304565	het	*TNFRSF4*	c.C28T	p.R10C	0.008007	22
8	rs12721593	het	*NBN*	c.C278T	p.S93L	0.000581	24.2
15	rs1352520577	het	*BLM*	c.A3334C	p.N1112H	0.0000088	26.5
16	rs11548656	het	*PLCG2*	c.A731G	p.H244R	0.02566	16.45
19	rs370053399	het	*STXBP2*	c.C601T	p.R201C	0.0004820	24.4
22	rs7264953	het	*TBX1*	c.G297A	p.A99A	0.02236	15.64
X	rs141756032	het	*CYBB*	c.G1090C	p.G364R	0.003974	23
Family Member 3 (female)							
1	rs202077872	het	*CR2*	c.G1117A	p.D373N	0.0005808	22.1
15	rs1352520577	het	*BLM*	c.A3334C	p.N1112H	0.0000088	26.5
16	rs11548656	het	*PLCG2*	c.A731G	p.H244R	0.02566	16.45
19	rs370053399	het	*STXBP2*	c.C601T	p.R201C	0.0004820	24.4
X	rs141756032	het	*CYBB*	c.G1090C	p.G364R	0.003974	23
Family Member 4 (male, carrier)							
15	rs1352520577	het	*BLM*	c.A3334C	p.N1112H	0.0000088	26.5
16	rs11548656	het	*PLCG2*	c.A731G	p.H244R	0.02566	16.45
19	rs370053399	het	*STXBP2*	c.C601T	p.R201C	0.0004820	24.4
22	rs7264953	het	*TBX1*	c.G297A	p.A99A	0.02236	15.64
Family Member 5 (male, SLE)							
1	rs35304565	het	*TNFRSF4*	c.C28T	p.R10C	0.008007	22
8	rs12721593	het	*NBN*	c.C278T	p.S93L	0.000581	24.2
Family Member 6 (male, aHUS)							
2	rs2066518	het	*SMARCAL1*	c.G1129C	p.E377Q	0.01031	24.9
7	rs3735131	het	*CARD11*	c.G2244C	p.T748T	0.02254	15.16
12	rs5745068	het	*POLE*	c.G6494A	p.R2165H	0.003786	33
16	rs3743930	het	*MEFV*	c.G442C	p.E148Q	0.01353	23.3
16	rs2066847	het	*NOD2*	c.3016_3017insC	p.A1007FS	0.015	35
16	rs11548656	het	*PLCG2*	c.A731G	p.H244R	0.02566	16.45
19	rs370053399	het	*STXBP2*	c.C601T	p.R201C	0.0004820	24.4
22	rs12484684	het	*IL17RA*	c.C1685A	p.P562Q	0.006	25.8

In an attempt to generalize our findings of the co-existence of additional non-*CD46* rare predicted functional relevant IEI-gene variants in *CD46* mutation carriers, we filtered the NIHR BioResource Rare Diseases project database for individuals carrying rare *CD46* variants and co-analyzed functionally likely relevant (CADD Score >15) rare non-*CD46* IEI-gene variants (Supplemental Table 4). With this approach we identified seven individuals carrying rare *CD46* variants already demonstrated to be associated with CD46 related disease [15]. In each of these individuals we identified up to ten additional rare, potentially functionally relevant variants in other IEI-related genes. These IEI-related non-*CD46* genes included genes linked to autoinflammation such as *ADA2* or *PLCG2* (Supplemental Table 4) [29]. Rare variants in complement system genes such as *C4A*, *C4B* or *C6* were also detected (Supplemental Table 4). In addition, four of the seven individuals carrying rare *CD46* missense variants also carried rare variants in *STAT5B*, of which gain of function mutations have been associated with autoinflammatory features [51].

## Discussion

The pediatric patient with aHUS has been followed at our institution for the last 17 years. Since his last flare in 2019 he is in good clinical condition without any medication. Renal function is normal without proteinuria. Due to the rarity of flares, there is currently no indication for prophylactic Eculizumab-mediated complement inhibition. The patient with CD46-associated SLE reported here has been followed at our clinic for more than 10 years. Clinically, the SLE initially manifested with toe ulceration due to anti-phospholipid syndrome, while other clinical manifestations, especially nephritis, did not (yet) develop. He has currently minor arthritis of the hands under hydroxychloroquine treatment. There is no current clinical manifestation of the anti-phospholipid syndrome under Aspirin treatment. To our knowledge, this is the first report that demonstrates association of a rare CD46 mutation with both aHUS and SLE in different individuals of the same family. The association of *CD46* mutations with SLE in the literature has not been studied in detail, neither at the clinical nor at the molecular level. SLE-associated *CD46* mutations have mostly not been functionally assessed [3].

In addition to its complement regulating function, CD46 has been recognized as a co-stimulator of T helper cells favoring Th1 induction [7, 10]. CD46 has also been demonstrated to be involved in T cell contraction *via* the timely co-induction of immune-regulative IL-10 secretion in successfully expanded Th1 cells [42]. So far, a more detailed analysis of the circulating T cell subpopulations in patients with *CD46* mutations has not been performed. Here, we demonstrate that most *CD46* mutation carriers, including healthy carriers, have low or low-normal naïve CD4^+^ T cell frequencies in peripheral blood. While complete lack of CD46 is accompanied by almost absent Th1 responses [7], our data here indicate that haploinsufficient CD46 expression may result in hyperactive T cells with less naïve and more memory T cells maintained/generated. Specifically, our finding that diseased *CD46* mutation carriers had reduced Treg frequencies may then skew the overall T cell activity towards being pathologically pro-inflammatory. Although we did not observe significant differences in cytokine production by carrier-derived T cells when activated in bulk, it should be noted that we were unable (due to limited sample volume) to carefully dissect the cytokine production of separate T cell subpopulations (for example T_CM_, T_EM_ etc.). We did not find consistent differences in T cell intrinsic CD46 expression in healthy *vs.* diseased mutation carriers, although we have not further defined the exact CD46 isoform expression profile. We have recently identified the C3–CD46 axis as a key characteristic of CD4^+^ and CD8^+^ T cells and macrophages in tissues [52]. It may therefore be interesting to assess tissue T cell activity in CD46-insufficient patients in the future. CD46-dependent metabolic adaptation following T cell activation was disturbed in both diseased and healthy mutation carriers with most prominent dysregulation in mitochondrial function, proportionate with decreased CD46 surface expression. We have recently demonstrated that genetically-determined mitochondrial dysfunction may indeed underlie chronic human immune-dysregulation [23].

None of the CD46 mutation carriers displayed an overt antibody deficiency in circulation although it has been reported that individuals with homozygous *CD46* mutations have common variable immunodeficiency (CVID)-like immunodeficiency [3]. We have, however, not measured antigen-specific B cell activation responses in this study.

Incomplete penetrance is commonly observed in IEI, even those that are highly mortal [53]. Several co-factors explaining incomplete penetrance in human inherited disease have been demonstrated: these include exogenous factors such as infections/vaccinations as well as genetic modifiers such as mutations in modifier genes [53, 54]. For CD46-associated disease, low clinical penetrance (at least in childhood) has even been described in patients with homozygous loss-of-expression *CD46* mutations [55]. This would be in keeping with the hypothesis of non-*CD46* rare immune gene alleles impacting on disease penetrance. Indeed, in-depth analysis of our diseased *CD46* mutation carriers assessed here showed that they carry private, rare and potentially functionally relevant (high CADD scores) variants in non-*CD46* IEI-related genes. The proinflammatory effects of the *MEFV* and *NOD2* variants found in the aHUS patient have been discussed above.

Polymorphisms in OX40/OX40L associated with enhanced protein expression have been described to be associated with SLE [56, 57] and human monosomy 1q36 evidences activated CD4^+^ T-cell sustainment via OX40 [58]. Like CD46, OX40 is a T cell co-stimulatory protein regulating T cell survival and is, similar to CD46, acting as an Akt-mTOR activator. It needs to be addressed in subsequent studies whether the Treg dysfunction described in CD46 mutated individuals [5] might be influenced by *TNFRSF4* variants. Effectively, Treg and follicular T helper cells have been demonstrated to be abnormal in human SLE in an OX40/OX40L-dependent manner [59, 60]. We do not demonstrate the p.R10C OX40 mutation to be gain-of-function. We however provide experimental evidence that CD4^+^ T cell-intrinsic OX40 expression is up-regulated by CD46 co-stimulation and that the SLE patient had augmented OX40 expression on CD4^+^ T cells ex vivo.

Epistasis has been described as the synergistic interaction of genetic loci potentially modifying disease phenotypes and severity. Epistatic interactions have been described for cystic fibrosis (*CFTR*) and *DCNT4* [61] or craniosynostosis (*SMAD6*) and *BMP2* amongst others [54, 62]. Within IEI, epistasis has been described for TNFRSF13B and TCF3 [63, 64]. It is increasingly acknowledged that clinical phenotypes of IEI with low penetrance are impacted by pathogenic mutations in other genes [53]. We extend this concept also beyond the familial *CD46* mutation carriers experimentally assessed here and show that individuals carrying rare, disease-causing *CD46* missense mutations may co-carry up to ten rare, likely functionally relevant mutations in other IEI genes, including genes driving autoinflammation or complement dysfunction [29]. Although these findings remain correlative, it is intriguing that some of the additional IEI-gene mutations found in our wider screen of patients with *CD46* mutations have previously been connected with CD46 activity: for example, C4 (encoded by the *C4A* gene) provides C4b as an (extrinsic or intrinsic) ligand for CD46 and CD46 is a strong STAT5 inducer in T cells [42, 65].

In summary, this is to our knowledge, the first study that correlates immune-phenotypic, immune-functional (including immune-metabolic) and genetic factors with clinical manifestations in carriers of a disease-associated *CD46* loss-of-expression mutation. This study suggests that assessment of peripheral T cell phenotype and activity as well as the search for rare genetic immune variants in immune-genes other than *CD46* may help to personalize the prognosis and treatment in patients carrying *CD46* and possibly other aHUS-related mutations.

### Supplementary Information

ESM 1 Supplemental Figure 1: a) Time of hospitalization, requirement of intensive care and duration of peritoneal dialysis during the five flares of aHUS. b) Laboratory parameters during the five flares of aHUS in family member number 6 (aHUS patient). Supplemental Figure 2: (Top) Clinical and laboratory manifestations of SLE in family member 5. X indicates presence of the criteria (Middle) Serum immunoglobulins in the SLE patient. (Bottom) Quantification of different anti-phospholipid autoantibodies Values in red are above the reference range. Supplemental Figure 3: a+b) The indicated lymphocyte and B cell subpopulations of all tested family members were assessed by flow-cytometry. c) The indicated T cell subpopulations in the SLE patient were repeated 6 years following the initial assessment. CM: central memory; EM: effector memory; TEMRA: terminally differentiated effector memory; RTE: recent thymic emigrants; fTh: follicular T helper; Treg: regulatory T cells; MHCII^+^: activated T cells. d) Quantification of the indicated serum immunoglobulins. e) Functional assessment of the indicated complement pathways using serum of the individual family members. The blue and green dots represent the aHUS and SLE patient, respectively. Closed symbols represent mutation carriers while the open symbol represents the family member lacking the mutated allele. The lines mark normal reference values. Supplemental Figure 4: Purified CD4^+^ T cells were left non-activated (NA) or stimulated with immobilized agonistic antibodies against CD3, CD3 + CD28 or CD3 + CD46. Supernatants were harvested 36 hours post stimulation and analyzed by cytometric bead array (CBA). Secretion of the indicated cytokines were normalized to anti-CD3-stimulated cells which was set to 1. All bars indicate means ± SD. The ratio of IFN-γ concentration divided by the IL-10 level in supernatants serves as a marker of proinflammatory CD4^+^ T cell function and is depicted in a separate graph. All bars indicate means ± SD. Analyzed by unpaired t-tests. Supplemental Figure 5: a) Multiple sequence alignment of human TNFRSF4/OX40 and its orthologues. The Arg10 residue of OX40 in humans and other species are colored in pink. b) Functional region prediction of TNFRSF4/OX40 via SignalP-6.0 (https://services.healthtech.dtu.dk/services/SignalP-6.0/). c) Representative TNFRSF4/OX40 expression profiles from one donor and quantification of five individual donors. CD4^+^ cells from healthy human donors were stimulated for 36h with the indicated antibody combinations. TNFRSF4/OX40 expression was measured on viable cells using anti-human CD134 (OX40) APC-conjugated mAb (ACT35). d) *Ex vivo* expression of OX40 (X axis) and 4-1BB (Y axis) on PBMC-derived viable CD4^+^ T cells of *CD46* mutation carrier 5 aligned with three healthy controls after 36-hour culture. Supplemental Table 1: Clinical history, as assessed with a standardized case report form, of all studied family members. Supplemental Table 2: a) Clinical manifestations of *CD46* mutation carrier 6 during the aHUS flares. b) Complement-related diagnostic-lab analysis during the aHUS flares. Supplemental Table 3: a) Autoantibody profiles of *CD46* mutation carrier 5 (SLE patient). b) Complement-related lab analysis in the SLE patient over time. Values in red are above the reference range, values in blue are below the reference range. Supplemental Table 4: The British NIHR BioResource Rare Diseases project database was filtered for individuals carrying rare (allele frequency <1/1000), likely functionally relevant (CADD Score >15) variants in CD46 which have already been described in patients with CD46 dependent disease. In such individuals, we searched for rare (allele frequency <1/1000), likely functionally relevant (CADD Score >15) variants in other IEI-related genes (defined as being included in the 2019 update on the IEI-classification from the International Union of Immunological Societies expert committee [29]. IEI genes were attributed to different clinical categories as proposed by the International Union of Immunological Societies (IUIS) [29]. Genes linked to autoinflammatory diseases are marked in green, genes linked to the function of the complement system are marked in blue. Mutations in *STAT5B* are marked in orange. (DOCX 766 kb)

ESM 2 (JPEG 24 kb)

ESM 3 (JPEG 36 kb)

## Data Availability

The datasets generated during and/or analyzed during the current study are available from the corresponding author on reasonable request.

## Abbreviations

IEI: inborn errors of immunity
PID: primary immunodeficiency
SLE: systemic lupus erythematosus
aHUS: atypical hemolytic uremic syndrome
TMA: thrombotic microangiopathy
PBMC: peripheral blood mononuclear cells
WES: whole exome sequencing
OCR: oxygen consumption rate
ECAR: extracellular acidification rate
