# Correction to: Immunologic and Genetic Contributors to CD46‑Dependent Immune Dysregulation

**DOI:** 10.1007/s10875-023-01563-y

**Published:** 2023-08-12

**Authors:** Benedikt J Meyer, Natalia Kunz, Sayuri Seki, Rebecca Higgins, Adhideb Ghosh, Robin Hupfer, Adrian Baldrich, Julia R Hirsiger, Annaïse J Jauch, Anne-Valerie Burgener, Jonas Lötscher, Markus Aschwanden, Michael Dickenmann, Mihaela Stegert, Christoph T Berger, Thomas Daikeler, Ingmar Heijnen, Alexander A Navarini, Christoph Rudin, Hiroyuki Yamamoto, Claudia Kemper, Christoph Hess, Mike Recher

**Affiliations:** 1 Immunodeficiency Laboratory, Department of Biomedicine, University Hospital Basel, Basel, Switzerland; 2 Immunobiology Laboratory, Department of Biomedicine, University Hospital Basel, Basel, Switzerland; 3 Complement and Inflammation Research Section, CIRS,DIR, NHLBI, NIH, Bethesda, USA; 4 AIDS Research Center, National Institute of Infectious Diseases, Tokyo, Japan; 5 Dermatology, University Hospital Basel, Basel, Switzerland; 6 Competence Center for Personalized Medicine, University of Zurich/Eidgenossische Technische Hochschule (ETH), Zurich, Switzerland; 7 Translational Immunology, Department of Biomedicine, University Hospital Basel, Basel, Switzerland; 8 Department of Angiology, University Hospital Basel, Basel, Switzerland; 9 Clinic for Transplantation Immunology and Nephrology, University Hospital Basel, Basel, Switzerland; 10 Rheumatology Clinic, University Hospital Basel, Basel, Switzerland; 11 University Center for Immunology, University Hospital Basel, Basel, Switzerland; 12 Division Medical Immunology, Laboratory Medicine, University Hospital Basel, Basel, Switzerland; 13 University Children’s Hospital, University of Basel, Basel, Switzerland; 14 Cambridge Institute of Therapeutic Immunology & Infectious Disease, Department of Medicine, University of Cambridge, Cambridge, UK

**Correction to: Journal of Clinical Immunology**

10.1007/s10875-023-01547-y

Due to typesetting mistake, the labels above the panel Figure 2B got duplicate data.

The original version has been corrected.

