# Supplementary material for: Immunologic and Genetic Contributors to CD46-Dependent Immune Dysregulation
Source: J Clin Immunol. 2023 Jul 21;43(8):1840–56. doi: 10.1007/s10875-023-01547-y (PMC10661731; doi:10.1007/s10875-023-01547-y)

Supplementary Materials

Supplemental Figure 1:

a) Time of hospitalization, requirement of intensive care and duration of peritoneal dialysis during the five flares of aHUS.

b) Laboratory parameters during the five flares of aHUS in family member number 6 (aHUS patient).

Supplemental Figure 2:

(Top) Clinical and laboratory manifestations of SLE in family member 5. X indicates presence of the criteria

(Middle) Serum immunoglobulins in the SLE patient.

(Bottom) Quantification of different anti-phospholipid autoantibodies

Values in red are above the reference range.

Supplemental Figure 3:

a+b) The indicated lymphocyte and B cell subpopulations of all tested family members were assessed by flow-cytometry.

c) The indicated T cell subpopulations in the SLE patient were repeated 6 years following the initial assessment. CM: central memory; EM: effector memory; TEMRA: terminally differentiated effector memory; RTE: recent thymic emigrants; fTh: follicular T helper; Treg: regulatory T cells; MHCII+: activated T cells.

d) Quantification of the indicated serum immunoglobulins.

e) Functional assessment of the indicated complement pathways using serum of the individual family members.

The blue and green dots represent the aHUS and SLE patient, respectively. Closed symbols represent mutation carriers while the open symbol represents the family member lacking the mutated allele. The lines mark normal reference values.

Supplemental Figure 4:

Purified CD4+ T cells were left non-activated (NA) or stimulated with immobilized agonistic antibodies against CD3, CD3 + CD28 or CD3 + CD46. Supernatants were harvested 36 hours post stimulation and analyzed by cytometric bead array (CBA). Secretion of the indicated cytokines were normalized to anti-CD3-stimulated cells which was set to 1. All bars indicate means ± SD. The ratio of IFN-γ concentration divided by the IL-10 level in supernatants serves as a marker of proinflammatory CD4+ T cell function and is depicted in a separate graph. All bars indicate means ± SD. Analyzed by unpaired t-tests.

Supplemental Figure 5:

a) Multiple sequence alignment of human TNFRSF4/OX40 and its orthologues. The Arg10 residue of OX40 in humans and other species are colored in pink.

b) Functional region prediction of TNFRSF4/OX40 via SignalP-6.0 (https://services.healthtech.dtu.dk/services/SignalP-6.0/).

c) Representative TNFRSF4/OX40 expression profiles from one donor and quantification of five individual donors. CD4+ cells from healthy human donors were stimulated for 36h with the indicated antibody combinations. TNFRSF4/OX40 expression was measured on viable cells using anti-human CD134 (OX40) APC-conjugated mAb (ACT35).

d) Ex vivo expression of OX40 (X axis) and 4-1BB (Y axis) on PBMC-derived viable CD4+ T cells of CD46 mutation carrier 5 aligned with three healthy controls after 36-hour culture.

Supplemental Table 1:

Clinical history, as assessed with a standardized case report form, of all studied family members.

Supplemental Table 2:

a) Clinical manifestations of CD46 mutation carrier 6 during the aHUS flares. b) Complement-related diagnostic-lab analysis during the aHUS flares.

Supplemental Table 3:

a) Autoantibody profiles of CD46 mutation carrier 5 (SLE patient).

b) Complement-related lab analysis in the SLE patient over time. Values in red are above the reference range, values in blue are below the reference range.

Supplemental Table 4:

The British NIHR BioResource Rare Diseases project database was filtered for individuals carrying rare (allele frequency <1/1000), likely functionally relevant (CADD Score >15) variants in CD46 which have already been described in patients with CD46 dependent disease. In such individuals, we searched for rare (allele frequency <1/1000), likely functionally relevant (CADD Score >15) variants in other IEI-related genes (defined as being included in the 2019 update on the IEI-classification from the International Union of Immunological Societies expert committee(29). IEI genes were attributed to different clinical categories as proposed by the International Union of Immunological Societies (IUIS)(29). Genes linked to autoinflammatory diseases are marked in green, genes linked to the function of the complement system are marked in blue. Mutations in STAT5B are marked in orange.


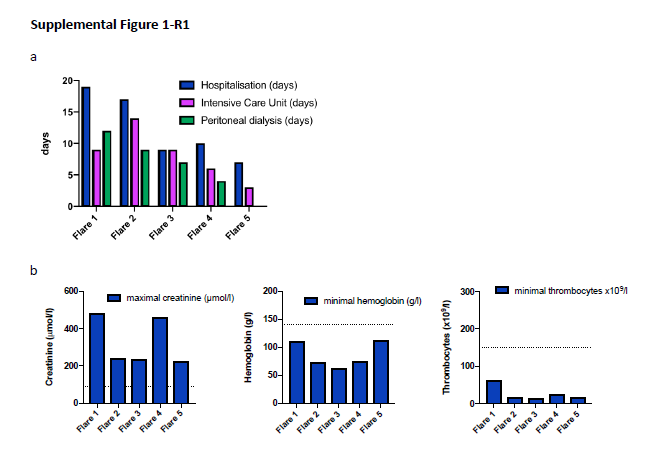


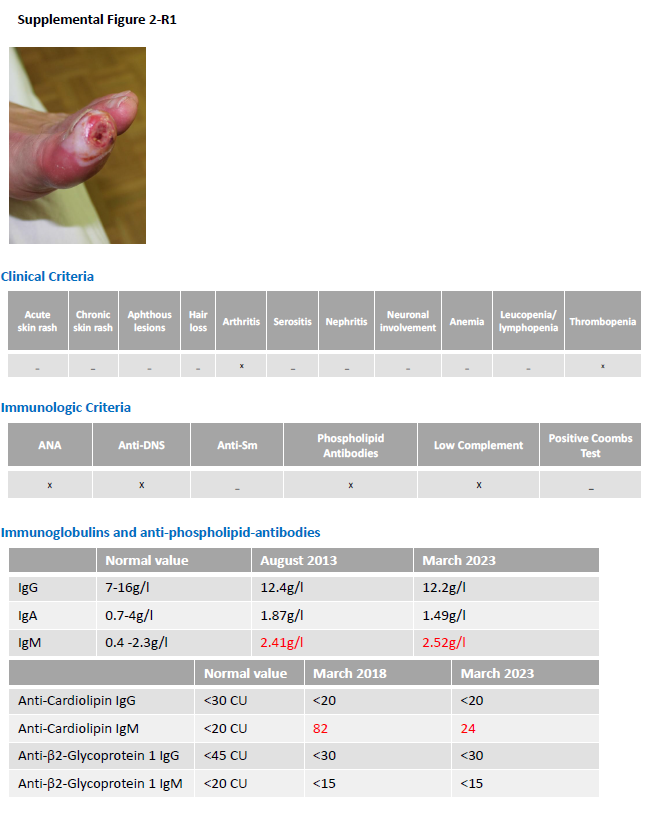


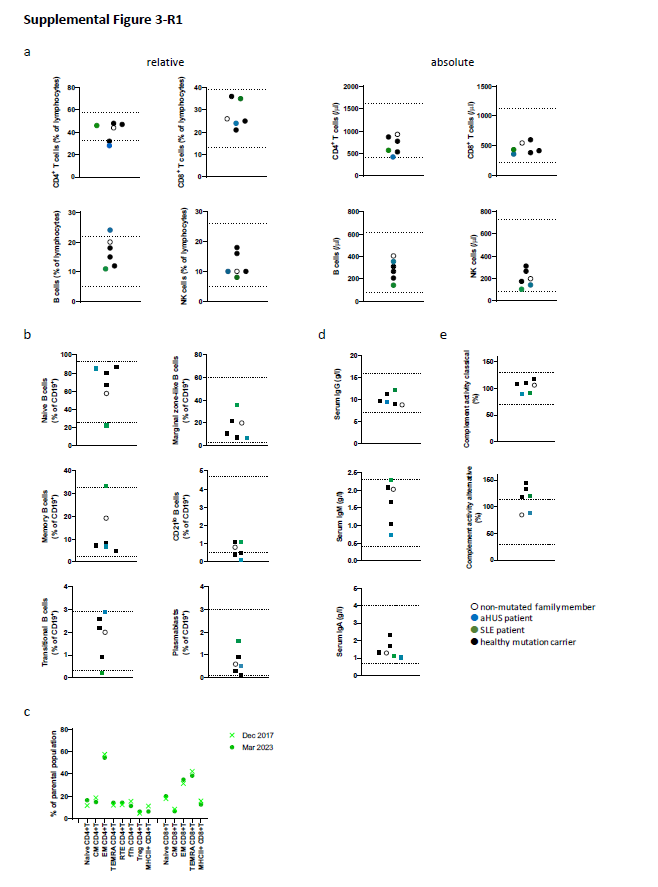


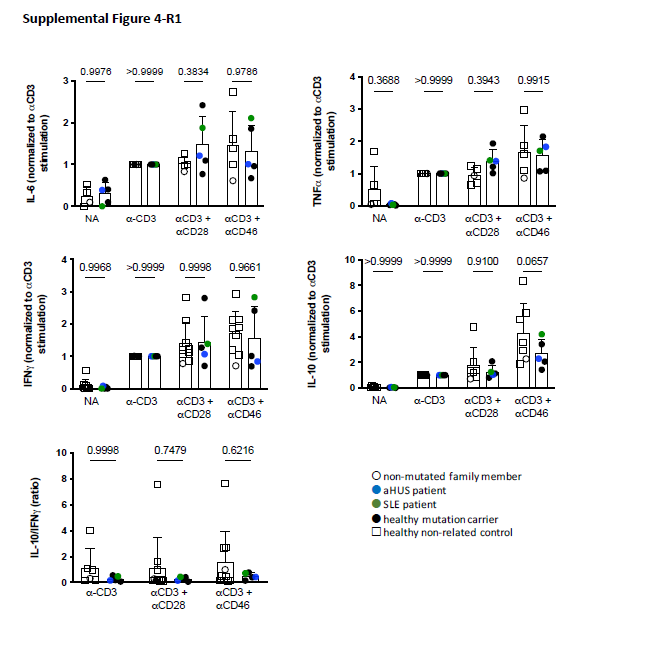


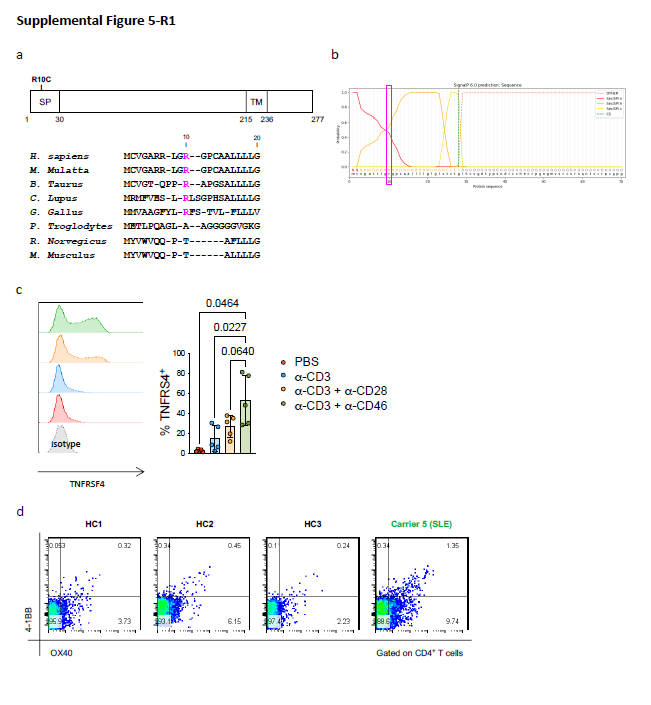


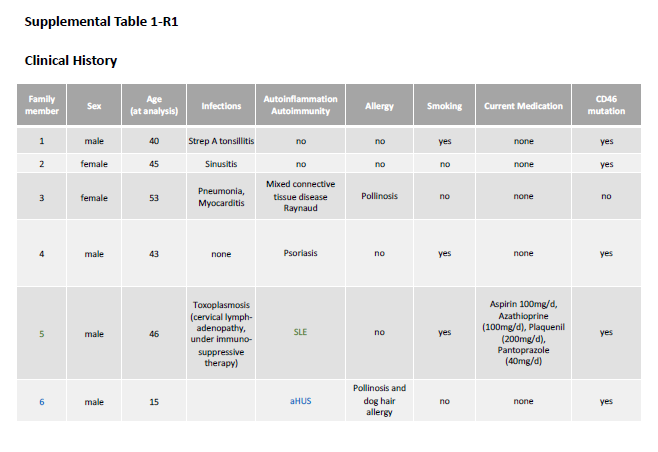


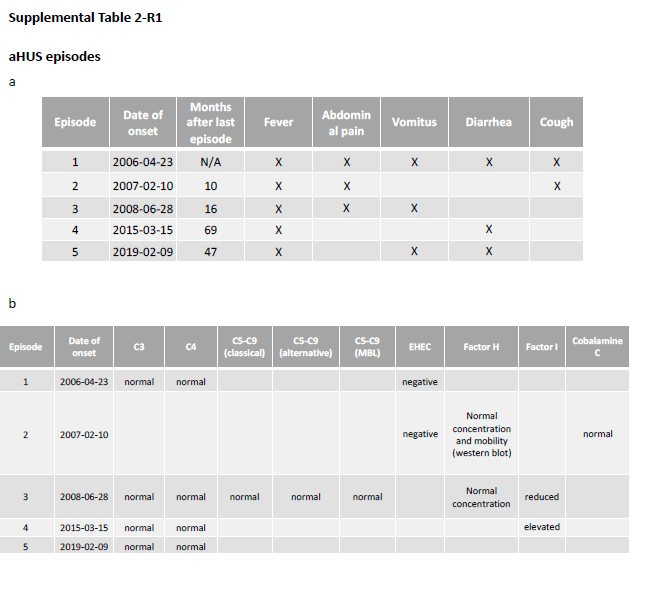


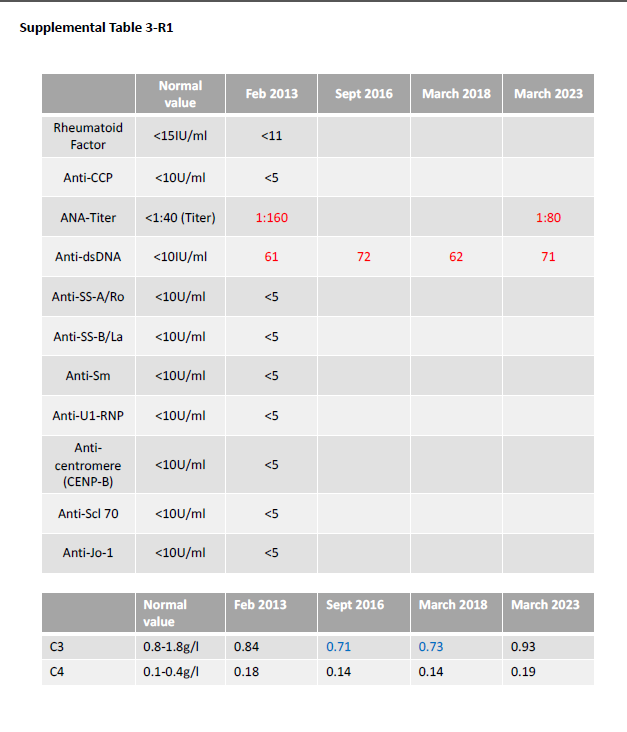


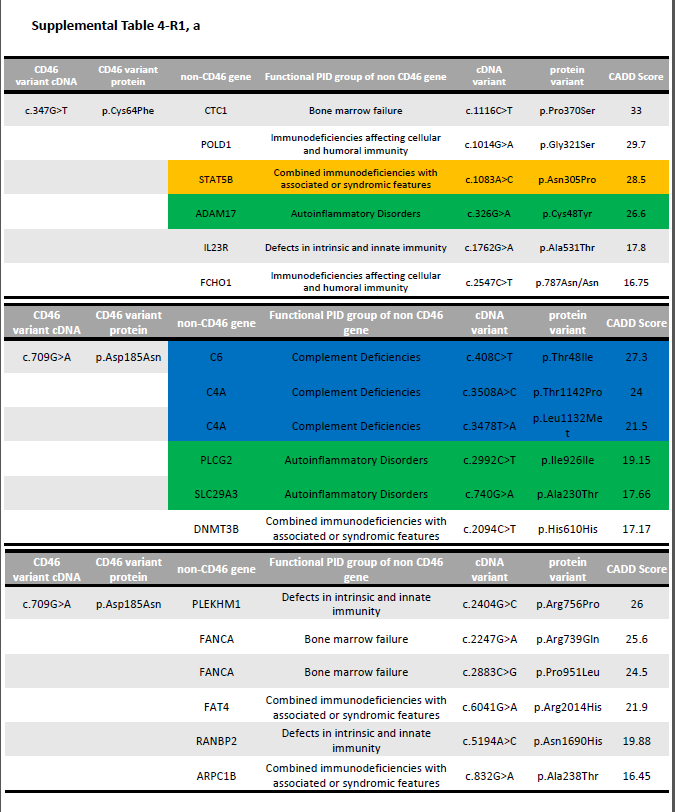


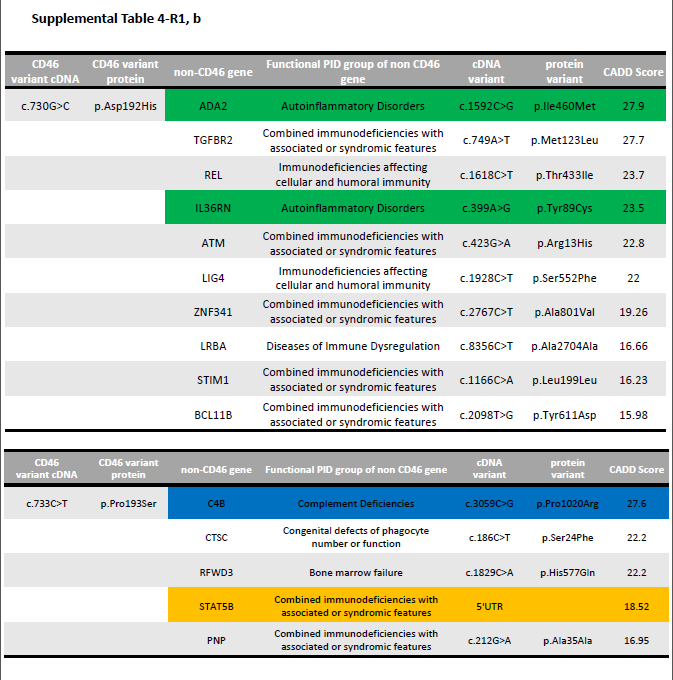


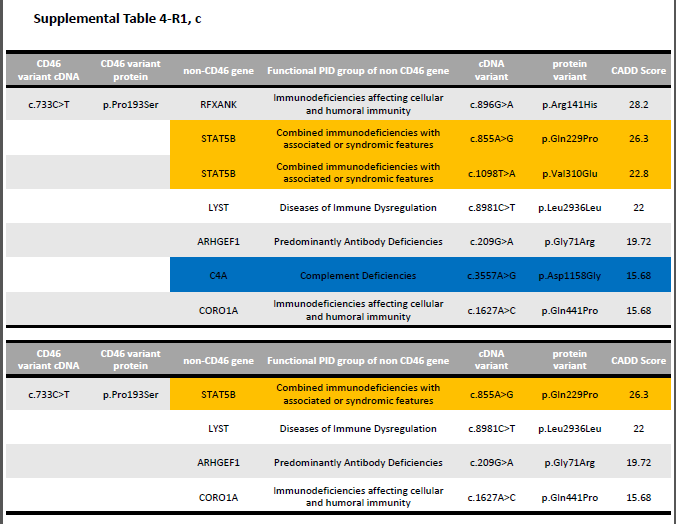

Supplement: Supplementary file 1 — Supplemental Figure 1: a) Time of hospitalization, requirement of intensive care and duration of peritoneal dialysis during the five flares of aHUS. b) Laboratory parameters during the five flares of aHUS in family member number 6 (aHUS patient). Supplemental Figure 2: (Top) Clinical and laboratory manifestations of SLE in family member 5. X indicates presence of the criteria (Middle) Serum immunoglobulins in the SLE patient. (Bottom) Quantification of different anti-phospholipid autoantibodies Values in red are above the reference range. Supplemental Figure 3: a+b) The indicated lymphocyte and B cell subpopulations of all tested family members were assessed by flow-cytometry. c) The indicated T cell subpopulations in the SLE patient were repeated 6 years following the initial assessment. CM: central memory; EM: effector memory; TEMRA: terminally differentiated effector memory; RTE: recent thymic emigrants; fTh: follicular T helper; Treg: regulatory T cells; MHCII+: activated T cells. d) Quantification of the indicated serum immunoglobulins. e) Functional assessment of the indicated complement pathways using serum of the individual family members. The blue and green dots represent the aHUS and SLE patient, respectively. Closed symbols represent mutation carriers while the open symbol represents the family member lacking the mutated allele. The lines mark normal reference values. Supplemental Figure 4: Purified CD4+ T cells were left non-activated (NA) or stimulated with immobilized agonistic antibodies against CD3, CD3 + CD28 or CD3 + CD46. Supernatants were harvested 36 hours post stimulation and analyzed by cytometric bead array (CBA). Secretion of the indicated cytokines were normalized to anti-CD3-stimulated cells which was set to 1. All bars indicate means ± SD. The ratio of IFN-γ concentration divided by the IL-10 level in supernatants serves as a marker of proinflammatory CD4+ T cell function and is depicted in a separate graph. All bars indicate means ± SD. [file 10875_2023_1547_MOESM1_ESM.docx]
