# Supplementary figures and images for: Immunologic and Genetic Contributors to CD46-Dependent Immune Dysregulation
Source: J Clin Immunol. 2023 Jul 21;43(8):1840–56. doi: 10.1007/s10875-023-01547-y (PMC10661731; doi:10.1007/s10875-023-01547-y)

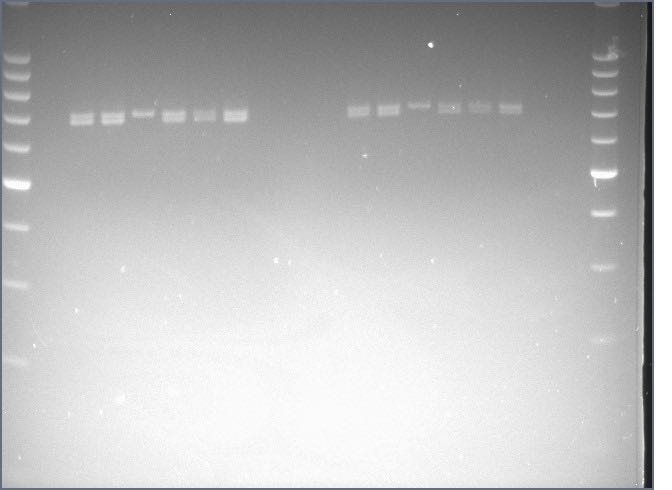

Supplement: Supplementary file 2 — (JPEG 24 kb) [file 10875_2023_1547_MOESM2_ESM.jpeg]

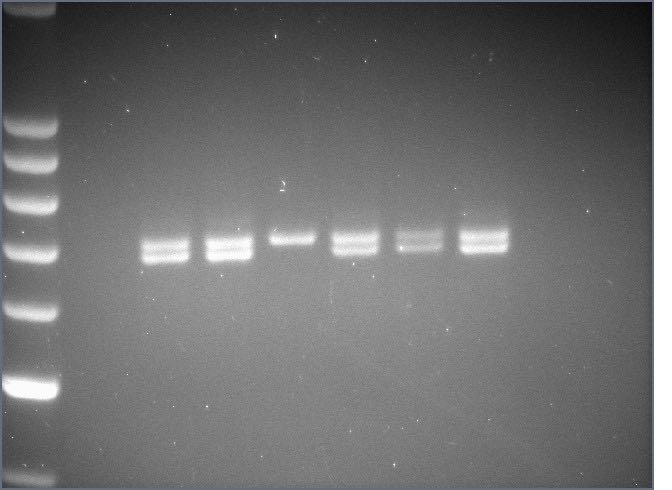

Supplement: Supplementary file 3 — (JPEG 36 kb) [file 10875_2023_1547_MOESM3_ESM.jpeg]
